# Dulaglutide improves muscle function by attenuating inflammation through OPA-1-TLR-9 signaling in aged mice

**DOI:** 10.18632/aging.203546

**Published:** 2021-09-19

**Authors:** Phyu Phyu Khin, Yeonhee Hong, MyeongHoon Yeon, Dae Ho Lee, Jong Han Lee, Hee-Sook Jun

**Affiliations:** 1 College of Pharmacy and Gachon Institute of Pharmaceutical Science, Gachon University, Incheon, Korea; 2 Lee Gil Ya Cancer and Diabetes Institute, Gachon University, Incheon, Korea; 3 Department of Internal Medicine, Gil Medical Center, Gachon University College of Medicine, Incheon, Korea; 4 Gachon Medical and Convergence Institute, Gil Medical Center, Incheon, Korea; 5 Department of Marine Bio and Medical Science, Hanseo University, Seosan, Korea

**Keywords:** glucagon-like peptide-1, dulaglutide, sarcopenia, optic atrophy-1, inflammation

## Abstract

Dulaglutide, a glucagon-like peptide-1 receptor (GLP-1R) agonist, is widely used to treat diabetes. However, its effects on muscle wasting due to aging are poorly understood. In the current study, we investigated the therapeutic potential and underlying mechanism of dulaglutide in muscle wasting in aged mice. Dulaglutide improved muscle mass and strength in aged mice. Histological analysis revealed that the cross-sectional area of the tibialis anterior (TA) in the dulaglutide-treated group was thicker than that in the vehicle group. Moreover, dulaglutide increased the shift toward middle and large-sized fibers in both young and aged mice compared to the vehicle. Dulaglutide increased myofiber type I and type IIa in young (18.5% and 8.2%) and aged (1.8% and 19.7%) mice, respectively, compared to the vehicle group. Peroxisome proliferator-activated receptor-gamma coactivator-1α (PGC-1α), a master regulator of mitochondrial biogenesis, decreased but increased by dulaglutide in aged mice. The expression of atrophic factors such as myostatin, atrogin-1, and muscle RING-finger protein-1 was decreased in aged mice, whereas that of the myogenic factor, MyoD, was increased in both young and aged mice following dulaglutide treatment. In aged mice, optic atrophy-1 (OPA-1) protein was decreased, whereas Toll-like receptor-9 (TLR-9) and its targeting inflammatory cytokines (interleukin-6 [IL-6] and tumor necrosis factor-α [TNF-α]) were elevated in the TA and quadriceps (QD) muscles. In contrast, dulaglutide administration reversed this expression pattern, thereby significantly attenuating the expression of inflammatory cytokines in aged mice. These data suggest that dulaglutide may exert beneficial effects in the treatment of muscle wasting due to aging.

## INTRODUCTION

Sarcopenia represents progressive loss of muscle mass, strength, and function with aging and is associated with adverse individual physical and metabolic changes [1]. This is a major threat to independent living for older adults [2]. In addition, sarcopenia imposes an economic burden on individual families as well as on public health and social care systems.

Although there are many contributors to the development of sarcopenia, elevated inflammation in the skeletal muscles is a predisposing factor [3]. Inflammation induces muscle wasting through direct and indirect catabolic effects [4, 5]. Mitochondrial dysfunction is the main cause of increased cellular oxidative stress, followed by muscle wasting via an inflammatory response [6, 7]. Mitochondrial dynamics are critical for maintaining mitochondrial function [7, 8]. Optic atrophy-1 (OPA-1) is an essential protein that promotes mitochondrial fusion [9]. In a mouse model of mitochondrial myopathy, overexpression of *Opa-1* protected against muscle loss and enhanced muscle function [10]. Conversely, specific deletion of *Opa-1* in muscles led to oxidative and endoplasmic reticulum stress, thereby inducing muscle atrophy [11]. Redox-sensitive transcription factors such as nuclear factor-kappa B (NF-κB) are directly involved in inflammatory processes [4, 12, 13]. In addition, mitochondrial DNA released from impaired mitochondria stimulates Toll-like receptor 9 (TLR-9) to induce inflammation [14].

Glucagon-like peptide-1 (GLP-1) is best known as an incretin hormone that restores glucose homeostasis by stimulating insulin secretion and elevating β-cell survival [15]. However, it also has a broader range of physiological actions through its receptor, GLP-1R [16–18]. GLP-1R is expressed in various tissues, including the pancreas, kidneys, and skeletal muscles [15, 19, 20]. In particular, the GLP-1R agonist, exendin-4, increases glucose uptake in the skeletal muscle of type 2 diabetic rats [21] as well as oxygen consumption and thermogenic gene expression in C2C12 muscle cells [22]. Recently, we reported that exendin-4 attenuates muscle atrophy in dexamethasone-induced muscle atrophy and chronic kidney disease-derived muscle atrophy models [23, 24].

In the current study, we examined whether dulaglutide, a long-acting GLP-1R agonist, attenuates muscle atrophy in an aged mouse model and investigated the underlying mechanism. Our studies demonstrated that dulaglutide administration attenuated muscle wasting and restored muscle strength by reducing inflammation through the OPA-1-TLR-9 signaling pathway in the tibialis anterior (TA) and quadriceps (QD) muscles of aged mice.

## RESULTS

### Dulaglutide increases muscle weight in aged mice

**Figure 1:**
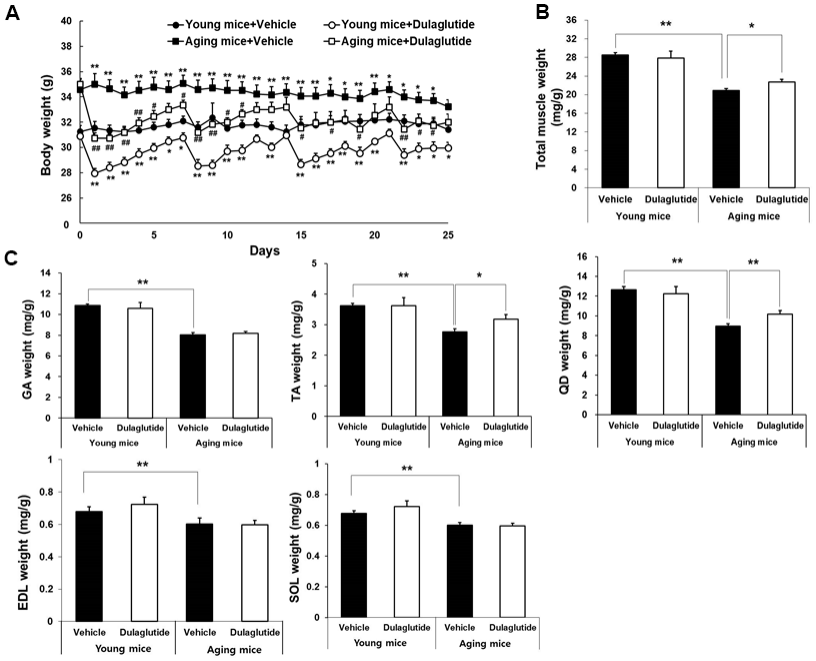
**Dulaglutide increases muscle weight in aged mice.** (**A**) Body weight changes in mice (**B**) Total muscle weight and, (**C**) Weight of the five muscle types, including gastrocnemius (GA), tibialis anterior (TA), quadriceps (QD), extensor digitorum longus (EDL), and soleus (SOL). The muscle weights were normalized to the body weight (g). The total muscle weight indicates the sum of all muscle weight such as SOL, TA, QD, GA, and EDL. All values are expressed as the mean ± SE. Significant differences are indicated as ** *p* < 0.01 or * *p* < 0.05 compared to young mice + vehicle or ## p < 0.01 or # p < 0.05 compared to aged mice + vehicle. N = 5–8/group.

The initial body weight was higher in aged mice compared to that in young mice, but decreased following dulaglutide treatment in both the age groups (Figure 1A). The weight loss was maximum on the first day following dulaglutide administration and then gradually recovered over time in both young and aged mice (Figure 1A). Total muscle mass was remarkably lower in the aged mice than in young mice. However, dulaglutide treatment significantly increased the total muscle mass in aged mice, but had no effect on the total muscle mass in young mice (Figure 1B). Consistent with the data on total muscle mass, the weights of the TA and QD muscles in aged mice increased following dulaglutide treatment without any change in the body weight at the end of the experiment (Figure 1C). However, this effect was not significant in any of the muscle types in the young mice (Figure 1C).

### Dulaglutide increases muscle strength in aged mice by increasing the size of myofibers

**Figure 2:**
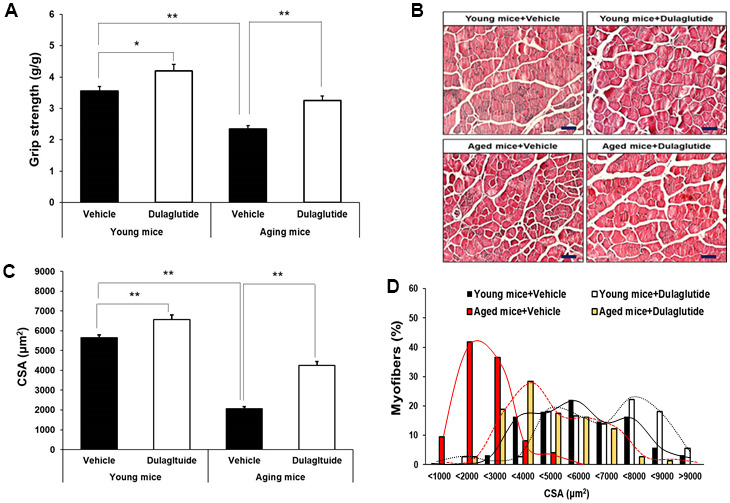
**Dulaglutide increases muscle function and myofiber size.** (**A**) Grip strengths were normalized to body weight (g). (**B**) Representative image of hematoxylin and eosin (H&E) stained muscle tissues (**C**) Cross-sectional area (CSA) of TA muscle tissue. Magnification ×200 (**D**) Muscle fiber size distribution. All values are expressed as the mean ± SE. Solid black line indicates young mice + vehicle, black dotted line indicates young mice + dulaglutide, solid red line indicates aged mice + vehicle, and red dotted line indicates aged mice + dulaglutide. Significant differences are indicated as ** *p* < 0.01 or * *p* < 0.05 compared to young mice + vehicle or aged mice + vehicle. N = 5–8/group.

Dulaglutide treatment significantly enhanced the grip strength in both age groups, although the effect was greater in aged mice than in young mice (Figure 2A). Interestingly, the improvement in grip strength in young mice was accomplished without an increase in the total muscle mass (Figure 2A). H&E staining of TA muscle was performed to better characterize the effect of dulaglutide in reversing muscle atrophy in aged mice. As with grip strength, the cross-sectional area (CSA) of muscle fibers was increased by dulaglutide in both young and aged mice groups compared to that of the vehicle groups (Figure 2B, 2C). In addition, dulaglutide treatment shifted the distribution of muscle fiber size from small to large fibers in both aged and young mice compared to vehicle groups (Figure 2D). Furthermore, triple staining of the gastrocnemius (GA) showed that dulaglutide administration increased myofiber type IIa by up to 18.5% and 1.8% in young and aged mice, respectively, compared to the vehicle group. This increase was much higher in young mice. In addition, the amounts of type I muscle fibers increased by up to 8.2% and 19.7% in young and aged mice, respectively (Supplementary Figure 1A, 1B). Peroxisome proliferator-activated receptor-gamma coactivator-1α (PGC-1α), a master regulator of mitochondrial biogenesis, was significantly lower in aged mice than in young mice. However, dulaglutide administration increased the expression of PGC-1α to the same extent as that in TA of young mice (Supplementary Figure 1C).

### Dulaglutide decreases the expression of muscle atrophic factors in aged mice

**Figure 3:**
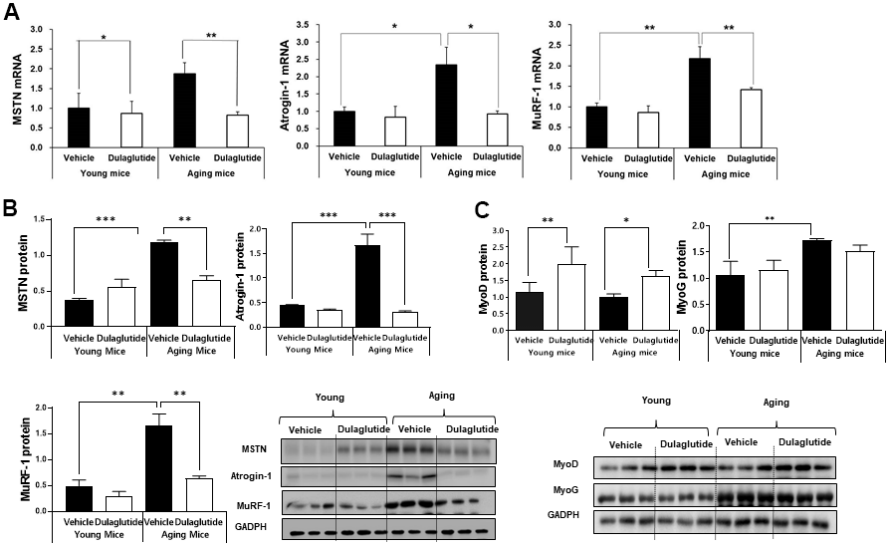
**Dulaglutide decreases muscle atrophic factors in aged mice.** (**A**) mRNA expression of myostatin (MSTN), atrogin-1, and muscle RING-finger protein-1 (MuRF-1) in the TA muscle (**B**) Protein levels of MSTN, atrogin-1, and MuRF-1 in the TA muscle (**C**) Protein level of myogenic factors, MyoD and myogenin (MyoG) in the TA muscle. All values are expressed as the mean ± SE. Significant differences are indicated as ***p < 0.001, ** *p* < 0.01, or * *p* < 0.05 compared to young mice + vehicle or aged mice + vehicle. N = 5–8/group.

To examine the changes in the expression of myogenic and muscle atrophic factors in TA muscle of aged mice following dulaglutide treatment, we evaluated the expression of myostatin (MSTN), atrogin-1, and muscle RING-finger protein-1 (MuRF-1). The mRNA and protein levels of muscle atrophic factors, including MSTN, atrogin-1, and MuRF-1, increased in the aged mice, but decreased following dulaglutide treatment (Figure 3A, 3B). Intriguingly, there was no change in the levels in the young mice (Figure 3A, 3B). In contrast, the mRNA levels of myogenic factors, MyoD and MyoG, were increased by dulaglutide in both aged and young mice (Supplementary Figure 2). While MyoD protein expression increased, there was no significant change in the protein expression of MyoG with dulaglutide treatment (Figure 3C).

### Dulaglutide attenuates inflammation and OPA-1-TLR-9 signaling pathway in aged mice

**Figure 4:**
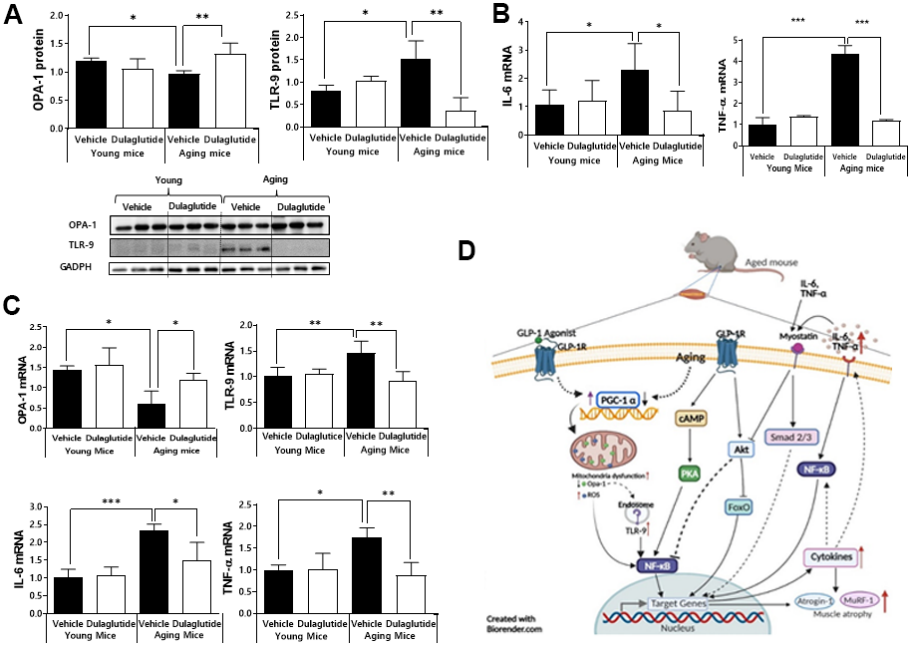
**Dulaglutide attenuates inflammation and downregulates the expression of OPA-1-TLR-9 signaling in aged mice.** (**A**) Protein levels of OPA-1 and TLR-9 in TA muscle (**B**) mRNA expression of IL-6 and TNF-α in TA muscle (**C**) mRNA expression of *Opa-1*, TLR-9, IL-6, and TNF-α in QD muscle. (**D**) Schematic diagram of the proposed mechanism of action of dulaglutide in muscle tissue. Our data suggest that dulaglutide mediated-GLP-1 receptor signaling may regulate muscle atrophy in aged mice via the following three independent and interconnected signaling pathways: 1) Dulaglutide stimulates PGC-1α, which in turn enhances mitochondrial biogenesis and function, subsequently suppressing endosomal TLR9 mediated NF-kB signaling cascades in muscle. This signaling involves an increase in proinflammatory cytokines and muscle atrophic factors expression. 2) The increase in proinflammatory cytokines activates myostatin signaling cascades and 3) NF-kB signaling cascades thereby inducing proinflammatory cytokines and muscle atrophic factors expression. In contrast, dulaglutide treatment suppresses these signaling pathways by regulating cAMP-PKA-NF-kB and AKT-FoxO. All values are expressed as the mean ± SE. Significant differences are indicated as ***p < 0.001, **p < 0.01, or *p < 0.05 compared to young mice + vehicle or aged mice + vehicle. N = 5–8/group.

Given that age-associated loss of *Opa-1* and upregulation of TLR-9 in muscle induce muscle atrophy [11, 25], we further investigated whether the expression of *Opa-1* and TLR-9 is regulated by dulaglutide. The expression of *Opa-1* was significantly decreased, whereas that of TLR-9 was increased in aged mice (Figure 4A). In contrast, dulaglutide administration reversed these expression patterns in the aged mice (Figure 4A). Notably, dulaglutide was not effective in the TA muscle in young mice (Figure 4A). The above finding led us to investigate whether expression of inflammatory cytokines was increased given the upregulation of TLR-9, because of its association with inflammation [26]. As expected, expression of IL-6 and TNF-α was elevated in aged mice compared to that in young mice. However, this increase was reduced following dulaglutide treatment in the TA muscle (Figure 4B). We further confirmed these findings in QD muscle, because of the small amount of TA muscle. As in the TA muscle, the expression of TLR-9 and other inflammatory cytokines (such as IL-6 and TNF-α) were significantly elevated in aged mice, but their expression was decreased by dulaglutide, unlike that of *Opa-1* (Figure 4C).

## DISCUSSION

We previously reported that the GLP-1R agonist, exendin-4, attenuates muscle atrophy in dexamethasone-induced and chronic kidney disease-derived muscle atrophy models [23]. Moreover, our recent study also demonstrated that dulaglutide treatment restores muscle fiber size, muscle mass, and muscle strength in a disuse muscle atrophy model [24]. In the current study, we further expanded our findings and proved that dulaglutide is also effective in age-induced muscle atrophy, as in sarcopenia. Mechanistically, dulaglutide treatment attenuated muscle atrophy in aged mice by decreasing OPA-1-TLR-9 mediated inflammatory responses and the E3 ubiquitin ligases, MuRF-1, and atrogin-1.

GLP-1 receptor agonists lower blood glucose levels by stimulating insulin secretion in the pancreas [27] and reducing food intake by slowing gastric emptying [28]. Thus, the weight loss observed on the first day post-dulaglutide administration was due to a reduction in food intake that was then gradually restored over time in the both young and aged mice in the present study.

Age reduces the total muscle mass and strength [29, 30]. In the case of aged gastrocnemius muscle, the ATP content is 50% lower than that in young animals, indicating that mitochondrial oxidative capacity decreases with age [31]. In the current study, the weight of all the muscle types assessed in aged mice was lower than that in young mice. Only TA and QD muscle mass significantly increased after 4 weeks of dulaglutide treatment, indicating that these two muscle types are the main contributors to the increase in total muscle mass. Intriguingly, dulaglutide did not affect the muscle mass of any of the muscle types in young mice, but elevated the grip strength in both groups. Aging-associated changes in muscle fiber composition are associated with mitochondrial number and function [32]. Therefore, it is possible that muscle types predominantly containing slow-type oxidative fibers, such as QD, are more effective than muscle types that mainly contain fast-type glycolytic fibers such as the extensor digitorum longus (EDL) and TA during aging. Similarly, exendin-4 has been shown to increase mitochondrial biogenesis, number, and mass in rat insulinoma cells [33], indicating that dulaglutide may directly affect the mitochondria and the degree of effect is influenced by mitochondrial content in each muscle type. Consistent with this report, although the amount of PGC-1α, a master regulator of mitochondrial biogenesis, was significantly decreased in aged mice, dulaglutide administration increased the expression of PGC-1α to the same extent as that in young mice in the TA muscle. Another alternative explanation may be the differences in the expression levels of GLP-1 receptors in individual muscle types. However, further studies are required to address these questions precisely. In addition, our immunofluorescence data further indicated that dulaglutide induced a muscle fiber switch from fast-type glycolytic fibers (type IIA, IIX, and IIB) to slow-type oxidative fibers (type I) in the GA muscle of aged mice. This may also enhance muscle strength and function without muscle mass. Taken together, these data indicate that dulaglutide may be involved not only in increasing the muscle mass, but also in the quality control of muscle tissues, such as via restoring mitochondrial function. The effect of dulaglutide on the quality control of muscle may be more effective in young mice, which further indicates that its effectiveness varies under different metabolic/physiologic conditions such as aging.

Histological analysis of the TA muscles showed that the size of the fibers in both young and aged mice increased following dulaglutide administration. In particular, small sized fibers (1000–3000 μm^2^) were predominant in aged mice compared to that in young mice. However, this distribution pattern shifted toward middle and large-sized fibers following dulaglutide administration, consistent with the average CSA, indicating that dulaglutide stimulates muscle cell differentiation and muscle fiber thickness associated with increased muscle strength. This observation is consistent with that in our previous study using disuse mice [24].

Aging is associated with an increase in the circulating levels of pro-inflammatory cytokines, such as IL-6 and TNF-α [34]. Several classical risk factors are regulated by these cytokines during muscle atrophy [35]. Studies have shown that administration of IL-6 or TNF-α in rats causes muscle breakdown, indicating that inflammation may be associated with the loss of muscle mass and muscle strength with aging [36]. MSTN, a negative regulator of muscle growth, induces muscle wasting through the regulation of MuRF-1 and atrogin-1 [37]. Our current data also showed that these muscle-specific E3 ligases were upregulated in aged mice, but inhibited following dulaglutide administration without any effect in young mice. In contrast, the mRNA expression of the myogenic factors was increased in both young and aged mice. However, we only observed an elevation in MyoD at the protein level in the current study, unlike that in our previous reports using different mouse models of muscle atrophy [23, 24]. Collectively, these data indicate that dulaglutide may regulate certain risk factors that drive E3 ligase expression under pathophysiological conditions.

Mitochondrial dysfunction is thought to play a critical role in the decline of muscle function [38]. OPA-1, a mitochondrial fusion protein, stabilizes mitochondrial DNA. In pathological conditions such as aging, mitochondrial alterations, such as a decrease in the total number or increase in oxidative damage, are increased in the skeletal muscle [39]. These biochemical and bioenergetic changes are due to a decrease in mitochondrial biogenesis and an increase in mitochondrial-mediated cell death [40–42]. Initial mitochondrial alterations activate TLR-9 by interacting with mtDNA, which induces or increases cellular inflammatory responses through activation of the TLR9/NF-κB pathway [13]. In particular, aging activates the TLR-9-MyD88-IL-6 pathway within the aorta in aged mice [11]. In skeletal muscle, ablation of *Opa-1* leads to severe mitochondrial inflammatory myopathy by activation of TLR-9 [26]. Similarly, our current data showed that TLR-9 is activated in aged mice, and that its expression is significantly inhibited by dulaglutide, indicating that dulaglutide may regulate inflammatory response through the OPA-1-TLR-9 signaling pathway.

In conclusion, we found that dulaglutide, a long-acting GLP-1R agonist, has therapeutic effects on muscle atrophy in aged mice by regulating the OPA-1-TLR-9 mediated inflammatory response and muscle-specific E3 ligase system (Figure 4D). These findings highlight the potential application of GLP-1R agonists in the treatment of aging associated muscle wasting.

## MATERIALS AND METHODS

### Animal models

Young (4-month-old) and old (24-month-old) C57BL/6J male mice were purchased from the Laboratory Animal Resource Center at the Korea Research Institute of Bioscience and Biotechnology. The mice were housed at ~23 ± 1° C under 12 h light/dark cycle on a standard laboratory diet. All experimental procedures were approved by the Institutional Animal Care and Use Committee of the Gachon University. The mice were subcutaneously administered 600 μg/kg/week dulaglutide (Trulicity®) for 4 weeks. Body weight and food intake were assessed daily. The mice were weighed and humanely sacrificed after 4 weeks of treatment. The skeletal muscles and other tissues were collected and weighed. All samples were stored at -80° C until use. For histological analysis, some parts of the muscle tissues were fixed in 10% neutral buffered formalin (NBF).

### Grip strength test

Limb grip strength was measured in mice using a grip strength meter (BIO-G53, BIOSEB, FL, USA), as described in our previous studies [23, 24].

### Immunohistochemistry

Serial transverse sections (8 μm) of paraffin embedded TA muscle samples were mounted on glass slides. Slides were stained with hematoxylin and eosin (H&E) and observed under a light microscope. The cross-sectional area (CSA) was assessed using Image J software.

### Multicolor immunofluorescent staining

The gastrocnemius (GA) muscles were immersed in an optimal cutting temperature solution immediately after dissection and frozen at −80° C. These optimal cutting temperature blocks were cut to a thickness of 10 μm. The tissues were fixed in 10% NBF for 20 min at room temperature and washed with phosphate-buffered saline. The fixed tissues were permeabilized at 25° C in phosphate-buffered saline containing 0.2% Triton X-100 for 40 min. The tissues were incubated with protein blocking solution (Dako, CA, USA) at 25° C for 1 h. To characterize the fiber type composition of GA muscles, tissues were immunolabeled with the following mouse monoclonal antibodies (Developmental Studies Hybridoma Bank [DSHB], USA): anti-type I (BA-F8, mouse IgG2b), anti-type IIA (SC-71, mouse IgG1), and anti-type IIB (BF-F3, mouse IgM). The sections were incubated overnight at 4° C in a mixture of BA-F8, SC-71, and BF-F3 antibodies (1:100). The sections were then incubated with secondary antibodies for 1 h at 25° C: DyLight 405 rabbit anti-mouse IgG2B (for BA-F8), Alexa Fluor 488-labeled goat anti-mouse IgG1 (for SC-71), and Alexa Fluor 555-labeled goat anti-mouse IgM (for BF-F3). Fluorescent images were taken at 100× magnification using a laser scanning confocal microscope (A1 plus, Nikon, Tokyo, Japan). Myofiber CSA was analyzed using ImageJ software. Blue labeled fibers were identified as type I, green labeled fibers as type IIA, red labeled fibers as type IIB, and unlabeled fibers as type IIX.

### Real time polymerase chain reaction (RT-PCR)

Gene expression was determined by quantitative RT-PCR (qRT-PCR) using SYBR Green PCR Master Mix (BIO-RAD system, USA), as described previously [23]. The primers used were as follows: TLR-9: 5'-ATG GTT CTC CGT CGA AGG ACT-3' (sense), 5'-GAG GCT TCA GCT CAC AGG G-3' (anti-sense), atrogin-1: 5'-GCA AAC ACT GCC ACA TTC TCT C-3' (sense), 5'-CTT GAG GGG AAA GTG AGA CG-3' (anti-sense), MuRF-1: 5'-TGA CCA CAG AGG GTA AAG-3' (sense), 5'-TGT CTC ACT CAT CTC CTT CTT C-3' (anti-sense), myostatin (MSTN): 5'-GGC CAT GAT CTT GCT GTA AC-3' (sense), 5'-TTG GGT GCG ATA ATC CAG TC-3' (anti-sense), *Opa-1*: 5'-ATA CTG GGA TCT GCT GTT GG-3' (sense), 5'-AAG TCA GGC ACA ATC CAC TT-3' (anti-sense), interleukin-6 (IL-6): 5'-TCC ATC CAG TTG CCT TCT-3' (sense), 5’-GGA GTG GTA TCC TCT GTG AA-3' (anti-sense), tumor necrosis factor-α (TNF-α): 5'-CCA ACG GCA TGG ATC TCA AAG ACA-3' (sense), 5'-TCC TCC CAT TCC AGG TAG GTG TTT-3' (anti-sense), cyclophilin: 5'-TGG AGA GCA CCA AGA CAG ACA-3' (sense), 5'-TGC CGG AGT CGA CAA TGA T-3' (anti-sense).

### Western blot analysis

Total protein was extracted from TA muscle tissue, and western blotting was performed as described previously [23]. The following antibodies were used: anti-MSTN (ab203076, Abcam, MA, USA), anti-MuRF-1(ab172479, Abcam), anti-atrogin-1 (ab74023), anti-myoblast determination protein 1 (MyoD) (sc-377460, Santa Cruz Biotechnology, CA, USA), anti-myogenin (sc-52903, Santa Cruz Biotechnology), anti-TLR-9 (NBP2-24729; Novus Biologicals), anti-OPA-1(#612606, BD Biosciences), anti-PGC-1α (ab54481, Abcam), and anti-GAPDH (sc-365062, Santa Cruz Biotechnology). The band density was quantified using ImageJ software and normalized to GAPDH.

### Statistical analysis

All statistical analyses were performed using one-way or two-way analysis of variance using the IBM SPSS Statistics 19 program (IBM, NY, USA). All values are expressed as mean ± standard error (SE). Statistical significance was set at P < 0.05.

### Data availability statement

Data are available from the corresponding author upon reasonable request.

## Supplementary Material

Supplementary Figures

## References

[1] Cruz-Jentoft AJ, Landi F, Schneider SM, Zúñiga C, Arai H, Boirie Y, Chen LK, Fielding RA, Martin FC, Michel JP, Sieber C, Stout JR, Studenski SA, et al. Prevalence of and interventions for sarcopenia in ageing adults: a systematic review. Report of the International Sarcopenia Initiative (EWGSOP and IWGS). Age Ageing. 2014; 43:748–59. 10.1093/ageing/afu115 25241753 PMC4204661

[2] Goisser S, Kemmler W, Porzel S, Volkert D, Sieber CC, Bollheimer LC, Freiberger E. Sarcopenic obesity and complex interventions with nutrition and exercise in community-dwelling older persons--a narrative review. Clin Interv Aging. 2015; 10:1267–82. 10.2147/CIA.S82454 26346071 PMC4531044

[3] Kudryavtseva AV, Krasnov GS, Dmitriev AA, Alekseev BY, Kardymon OL, Sadritdinova AF, Fedorova MS, Pokrovsky AV, Melnikova NV, Kaprin AD, Moskalev AA, Snezhkina AV. Mitochondrial dysfunction and oxidative stress in aging and cancer. Oncotarget. 2016; 7:44879–905. 10.18632/oncotarget.9821 27270647 PMC5216692

[4] Li H, Malhotra S, Kumar A. Nuclear factor-kappa B signaling in skeletal muscle atrophy. J Mol Med (Berl). 2008; 86:1113–26. 10.1007/s00109-008-0373-8 18574572 PMC2597184

[5] Cai D, Frantz JD, Tawa NE Jr, Melendez PA, Oh BC, Lidov HG, Hasselgren PO, Frontera WR, Lee J, Glass DJ, Shoelson SE. IKKbeta/NF-kappaB activation causes severe muscle wasting in mice. Cell. 2004; 119:285–98. 10.1016/j.cell.2004.09.027 15479644

[6] Georgieva E, Ivanova D, Zhelev Z, Bakalova R, Gulubova M, Aoki I. Mitochondrial Dysfunction and Redox Imbalance as a Diagnostic Marker of “Free Radical Diseases”. Anticancer Res. 2017; 37:5373–81. 10.21873/anticanres.11963 28982845

[7] Romanello V, Sandri M. Mitochondrial Quality Control and Muscle Mass Maintenance. Front Physiol. 2016; 6:422. 10.3389/fphys.2015.00422 26793123 PMC4709858

[8] Chen H, Vermulst M, Wang YE, Chomyn A, Prolla TA, McCaffery JM, Chan DC. Mitochondrial fusion is required for mtDNA stability in skeletal muscle and tolerance of mtDNA mutations. Cell. 2010; 141:280–89. 10.1016/j.cell.2010.02.026 20403324 PMC2876819

[9] Romanello V, Scalabrin M, Albiero M, Blaauw B, Scorrano L, Sandri M. Inhibition of the Fission Machinery Mitigates OPA1 Impairment in Adult Skeletal Muscles. Cells. 2019; 8:597. 10.3390/cells8060597 31208084 PMC6627087

[10] Civiletto G, Varanita T, Cerutti R, Gorletta T, Barbaro S, Marchet S, Lamperti C, Viscomi C, Scorrano L, Zeviani M. Opa1 overexpression ameliorates the phenotype of two mitochondrial disease mouse models. Cell Metab. 2015; 21:845–54. 10.1016/j.cmet.2015.04.016 26039449 PMC4457891

[11] Tezze C, Romanello V, Desbats MA, Fadini GP, Albiero M, Favaro G, Ciciliot S, Soriano ME, Morbidoni V, Cerqua C, Loefler S, Kern H, Franceschi C, et al. Age-Associated Loss of OPA1 in Muscle Impacts Muscle Mass, Metabolic Homeostasis, Systemic Inflammation, and Epithelial Senescence. Cell Metab. 2017; 25:1374–89.e6. 10.1016/j.cmet.2017.04.021 28552492 PMC5462533

[12] Lavrovsky Y, Chatterjee B, Clark RA, Roy AK. Role of redox-regulated transcription factors in inflammation, aging and age-related diseases. Exp Gerontol. 2000; 35:521–32. 10.1016/s0531-5565(00)00118-2 10978675

[13] Zhang JZ, Liu Z, Liu J, Ren JX, Sun TS. Mitochondrial DNA induces inflammation and increases TLR9/NF-κB expression in lung tissue. Int J Mol Med. 2014; 33:817–24. 10.3892/ijmm.2014.1650 24535292 PMC3976143

[14] Oka T, Hikoso S, Yamaguchi O, Taneike M, Takeda T, Tamai T, Oyabu J, Murakawa T, Nakayama H, Nishida K, Akira S, Yamamoto A, Komuro I, Otsu K. Mitochondrial DNA that escapes from autophagy causes inflammation and heart failure. Nature. 2012; 485:251–55. 10.1038/nature10992 22535248 PMC3378041

[15] Rowlands J, Heng J, Newsholme P, Carlessi R. Pleiotropic Effects of GLP-1 and Analogs on Cell Signaling, Metabolism, and Function. Front Endocrinol (Lausanne). 2018; 9:672. 10.3389/fendo.2018.00672 30532733 PMC6266510

[16] Meloni AR, DeYoung MB, Lowe C, Parkes DG. GLP-1 receptor activated insulin secretion from pancreatic β-cells: mechanism and glucose dependence. Diabetes Obes Metab. 2013; 15:15–27. 10.1111/j.1463-1326.2012.01663.x 22776039 PMC3556522

[17] Edwards CM, Todd JF, Mahmoudi M, Wang Z, Wang RM, Ghatei MA, Bloom SR. Glucagon-like peptide 1 has a physiological role in the control of postprandial glucose in humans: studies with the antagonist exendin 9-39. Diabetes. 1999; 48:86–93. 10.2337/diabetes.48.1.86 9892226

[18] Drucker DJ. The biology of incretin hormones. Cell Metab. 2006; 3:153–65. 10.1016/j.cmet.2006.01.004 16517403

[19] Tornehave D, Kristensen P, Rømer J, Knudsen LB, Heller RS. Expression of the GLP-1 receptor in mouse, rat, and human pancreas. J Histochem Cytochem. 2008; 56:841–51. 10.1369/jhc.2008.951319 18541709 PMC2516959

[20] Fujita H, Morii T, Fujishima H, Sato T, Shimizu T, Hosoba M, Tsukiyama K, Narita T, Takahashi T, Drucker DJ, Seino Y, Yamada Y. The protective roles of GLP-1R signaling in diabetic nephropathy: possible mechanism and therapeutic potential. Kidney Int. 2014; 85:579–89. 10.1038/ki.2013.427 24152968

[21] Arnés L, Moreno P, Nuche-Berenguer B, Valverde I, Villanueva-Peñacarrillo ML. Effect of exendin-4 treatment upon glucose uptake parameters in rat liver and muscle, in normal and type 2 diabetic state. Regul Pept. 2009; 153:88–92. 10.1016/j.regpep.2008.08.005 18804493

[22] Choung JS, Lee YS, Jun HS. Exendin-4 increases oxygen consumption and thermogenic gene expression in muscle cells. J Mol Endocrinol. 2017; 58:79–90. 10.1530/JME-16-0078 27872157

[23] Hong Y, Lee JH, Jeong KW, Choi CS, Jun HS. Amelioration of muscle wasting by glucagon-like peptide-1 receptor agonist in muscle atrophy. J Cachexia Sarcopenia Muscle. 2019; 10:903–18. 10.1002/jcsm.12434 31020810 PMC6711418

[24] Nguyen TT, Choi H, Jun HS. Preventive Effects of Dulaglutide on Disuse Muscle Atrophy Through Inhibition of Inflammation and Apoptosis by Induction of Hsp72 Expression. Front Pharmacol. 2020; 11:90. 10.3389/fphar.2020.00090 32153405 PMC7046759

[25] Lyu AK, Zhu SY, Chen JL, Zhao YX, Pu D, Luo C, Lyu Q, Fan Z, Sun Y, Wu J, Zhao KX, Xiao Q. Inhibition of TLR9 attenuates skeletal muscle fibrosis in aged sarcopenic mice via the p53/SIRT1 pathway. Exp Gerontol. 2019; 122:25–33. 10.1016/j.exger.2019.04.008 31003004

[26] Rodríguez-Nuevo A, Díaz-Ramos A, Noguera E, Díaz-Sáez F, Duran X, Muñoz JP, Romero M, Plana N, Sebastián D, Tezze C, Romanello V, Ribas F, Seco J, et al. Mitochondrial DNA and TLR9 drive muscle inflammation upon Opa1 deficiency. EMBO J. 2018; 37:e96553. 10.15252/embj.201796553 29632021 PMC5978453

[27] MacDonald PE, El-Kholy W, Riedel MJ, Salapatek AM, Light PE, Wheeler MB. The multiple actions of GLP-1 on the process of glucose-stimulated insulin secretion. Diabetes. 2002 (Suppl 3); 51:S434–42. 10.2337/diabetes.51.2007.s434 12475787

[28] Tong J, D’Alessio D. Give the receptor a brake: slowing gastric emptying by GLP-1. Diabetes. 2014; 63:407–09. 10.2337/db13-1764 24464721

[29] Palomero J, Vasilaki A, Pye D, McArdle A, Jackson MJ. Aging increases the oxidation of dichlorohydrofluorescein in single isolated skeletal muscle fibers at rest, but not during contractions. Am J Physiol Regul Integr Comp Physiol. 2013; 305:R351–58. 10.1152/ajpregu.00530.2012 23697797 PMC3833391

[30] Sayer AA, Robinson SM, Patel HP, Shavlakadze T, Cooper C, Grounds MD. New horizons in the pathogenesis, diagnosis and management of sarcopenia. Age Ageing. 2013; 42:145–50. 10.1093/ageing/afs191 23315797 PMC3575121

[31] Drew B, Phaneuf S, Dirks A, Selman C, Gredilla R, Lezza A, Barja G, Leeuwenburgh C. Effects of aging and caloric restriction on mitochondrial energy production in gastrocnemius muscle and heart. Am J Physiol Regul Integr Comp Physiol. 2003; 284:R474–80. 10.1152/ajpregu.00455.2002 12388443

[32] Crupi AN, Nunnelee JS, Taylor DJ, Thomas A, Vit JP, Riera CE, Gottlieb RA, Goodridge HS. Oxidative muscles have better mitochondrial homeostasis than glycolytic muscles throughout life and maintain mitochondrial function during aging. Aging (Albany NY). 2018; 10:3327–52. 10.18632/aging.101643 30449736 PMC6286850

[33] Jung HS. Clinical Implications of Glucose Variability: Chronic Complications of Diabetes. Endocrinol Metab (Seoul). 2015; 30:167–74. 10.3803/EnM.2015.30.2.167 26194076 PMC4508260

[34] Bruunsgaard H. Effects of tumor necrosis factor-alpha and interleukin-6 in elderly populations. Eur Cytokine Netw. 2002; 13:389–91. 12517724

[35] Howard EE, Pasiakos SM, Blesso CN, Fussell MA, Rodriguez NR. Divergent Roles of Inflammation in Skeletal Muscle Recovery From Injury. Front Physiol. 2020; 11:87. 10.3389/fphys.2020.00087 32116792 PMC7031348

[36] Goodman MN. Interleukin-6 induces skeletal muscle protein breakdown in rats. Proc Soc Exp Biol Med. 1994; 205:182–85. 10.3181/00379727-205-43695 8108469

[37] Thomas M, Langley B, Berry C, Sharma M, Kirk S, Bass J, Kambadur R. Myostatin, a negative regulator of muscle growth, functions by inhibiting myoblast proliferation. J Biol Chem. 2000; 275:40235–43. 10.1074/jbc.M004356200 10976104

[38] Payne BA, Chinnery PF. Mitochondrial dysfunction in aging: Much progress but many unresolved questions. Biochim Biophys Acta. 2015; 1847:1347–53. 10.1016/j.bbabio.2015.05.022 26050973 PMC4580208

[39] Sun N, Youle RJ, Finkel T. The Mitochondrial Basis of Aging. Mol Cell. 2016; 61:654–66. 10.1016/j.molcel.2016.01.028 26942670 PMC4779179

[40] Peterson CM, Johannsen DL, Ravussin E. Skeletal muscle mitochondria and aging: a review. J Aging Res. 2012; 2012:194821. 10.1155/2012/194821 22888430 PMC3408651

[41] Gomes MJ, Martinez PF, Pagan LU, Damatto RL, Cezar MD, Lima AR, Okoshi K, Okoshi MP. Skeletal muscle aging: influence of oxidative stress and physical exercise. Oncotarget. 2017; 8:20428–40. 10.18632/oncotarget.14670 28099900 PMC5386774

[42] Szentesi P, Csernoch L, Dux L, Keller-Pintér A. Changes in Redox Signaling in the Skeletal Muscle with Aging. Oxid Med Cell Longev. 2019; 2019:4617801. 10.1155/2019/4617801 30800208 PMC6360032

