# Supplementary material for: Dulaglutide improves muscle function by attenuating inflammation through OPA-1-TLR-9 signaling in aged mice
Source: Aging (Albany NY). 2021 Sep 19;13(18):21962–74. doi: 10.18632/aging.203546 (PMC8507261; doi:10.18632/aging.203546)
Supplement: Supplementary Figures [file aging-13-203546-s001.pdf]

SUPPLEMENTARY FIGURES

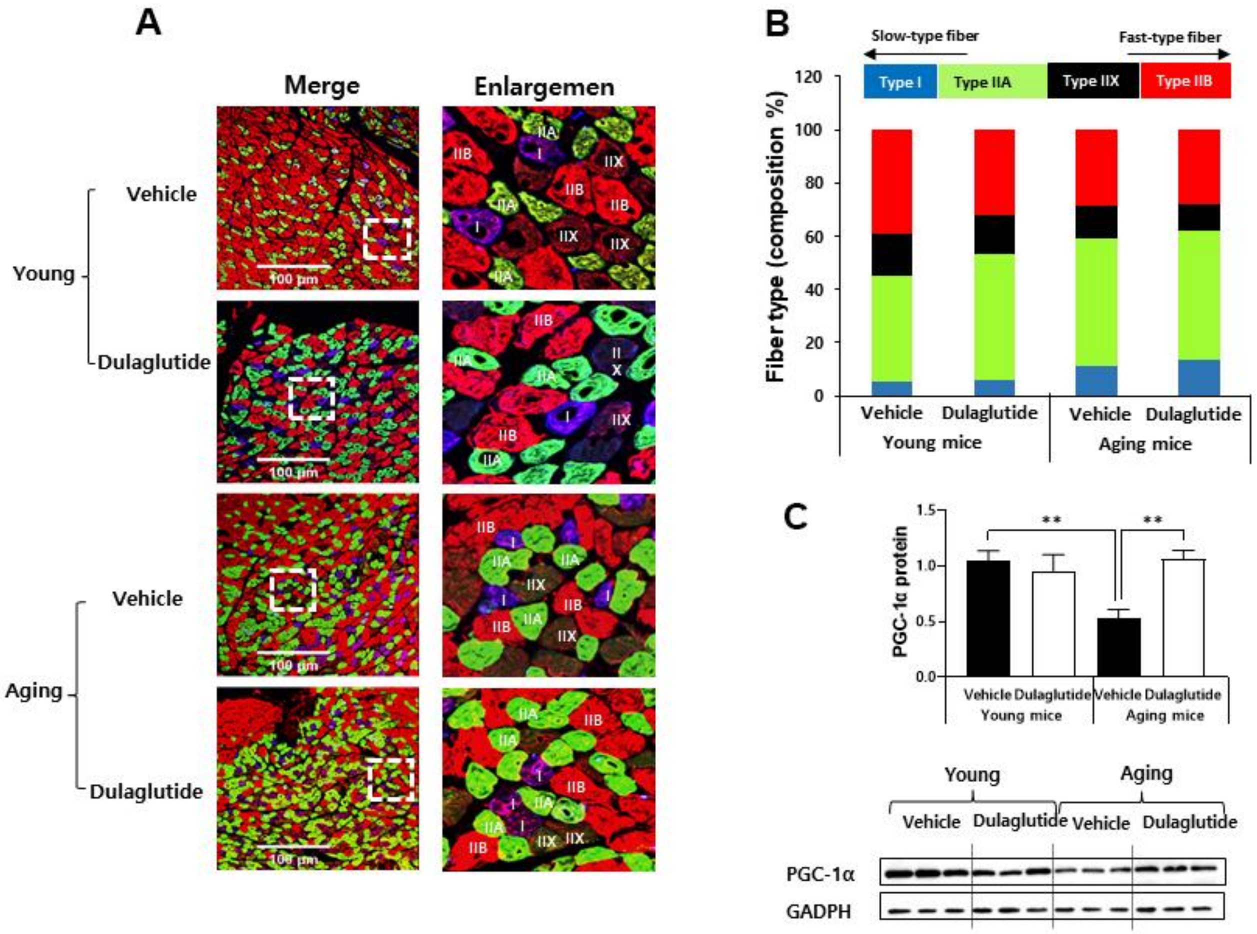

Supplementary Figure 1. Changes in myosin heavy chain (MyHC) expression by dulaglutide treatment. (**A**) Representative images of MyHC immunofluorescence in gastrocnemius muscle tissues for the triple-labeling with MyHC-I (blue, Type I fibers), MyHC-IIA (green, Type IIa fibers), and MyHC-IIB (red, type IIb fibers). (**B**) Proportion of MyHC isoforms-specific fibers. (**C**) Protein levels of PGC-1α in the TA muscle. The data are presented as the mean ± SE. *$p < 0.05$, **$p < 0.01$. N = 4 /group.

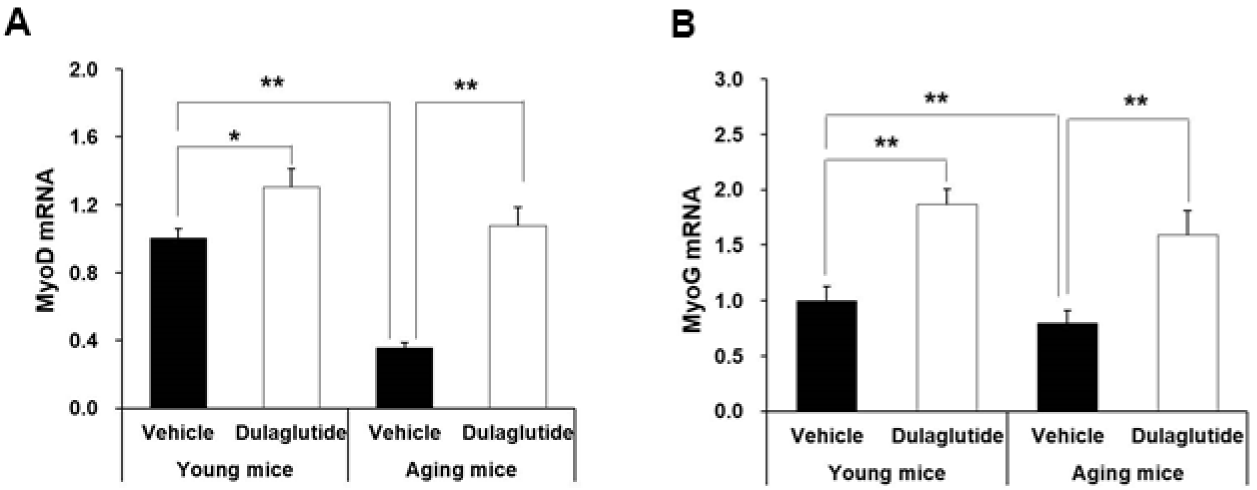

Supplementary Figure 2. Dulaglutide treatment increases myogenic factors in both young and aged mice. (**A**) mRNA expression of MyoD in the TA muscle (**B**) mRNA expression of MyoG in the TA muscle. All values are expressed as the mean ± SE. Significant differences are indicated as **p < 0.01, or *p < 0.05 compared to young mice + vehicle or aged mice + vehicle. N = 5–8/group.
